# Methodological Framework for Estimating the Correlation Dimension in HRV Signals

**DOI:** 10.1155/2014/129248

**Published:** 2014-01-30

**Authors:** Juan Bolea, Pablo Laguna, José María Remartínez, Eva Rovira, Augusto Navarro, Raquel Bailón

**Affiliations:** ^1^Communications Technology Group (GTC), Aragón Institute for Engineering Research (I3A), IIS Aragón, University of Zaragoza, 50018 Zaragoza, Spain; ^2^CIBER de Bioingeniería, Biomateriales y Nanomedicina (CIBER-BBN), 50018 Zaragoza, Spain; ^3^Anaesthesiology Service, Miguel Servet University Hospital, 50009 Zaragoza, Spain; ^4^Medicine School, University of Zaragoza, 50009 Zaragoza, Spain; ^5^Aragón Health Sciences Institute (IACS), 50009 Zaragoza, Spain

## Abstract

This paper presents a methodological framework for robust estimation of the correlation dimension in HRV signals. It includes (i) a fast algorithm for on-line computation of correlation sums; (ii) log-log curves fitting to a sigmoidal function for robust maximum slope estimation discarding the estimation according to fitting requirements; (iii) three different approaches for linear region slope estimation based on latter point; and (iv) exponential fitting for robust estimation of saturation level of slope series with increasing embedded dimension to finally obtain the correlation dimension estimate. Each approach for slope estimation leads to a correlation dimension estimate, called ${\hat{D}}_{2}$, ${\hat{D}}_{2\left(⊥\right)}$, and ${\hat{D}}_{2\left(\text{max}\right)}$. ${\hat{D}}_{2}$ and ${\hat{D}}_{2\left(\text{max}\right)}$ estimate the theoretical value of correlation dimension for the Lorenz attractor with relative error of 4%, and ${\hat{D}}_{2\left(⊥\right)}$ with 1%. The three approaches are applied to HRV signals of pregnant women before spinal anesthesia for cesarean delivery in order to identify patients at risk for hypotension. ${\hat{D}}_{2}$ keeps the 81% of accuracy previously described in the literature while ${\hat{D}}_{2\left(⊥\right)}$ and ${\hat{D}}_{2\left(\text{max}\right)}$ approaches reach 91% of accuracy in the same database.

## 1. Introduction 

Heart rate variability (HRV) has been widely used as a marker of the autonomic nervous system (ANS) regulation of the heart. Classical HRV indices include global descriptive statistics which characterize HRV distribution in the time domain (mean heart rate and standard deviation of the normal-to-normal beat interval, among others) and in the frequency domain (power in the very low frequency, and low frequency (LF) and high frequency (HF) bands). The activity of the two main branches of the ANS, sympathetic and parasympathetic systems, has been related with the power in the LF and HF bands, respectively [1].

HRV data often present nonlinear characteristics, possibly reflecting intrinsic physiological nonlinearities, such as changes in the gain of baroreflex feedback loops or delays in conduction time, which are not properly described by classical HRV indices.

The most widespread methods used to characterize nonlinear system dynamics are based on chaos theory. The question of whether HRV arises from a low-dimensional attractor associated with a deterministic nonlinear dynamical system or whether it has a stochastic origin is still under debate.

Of great interest is the concept of system complexity, which refers to the richness of process dynamics. Complexity measures are based on the theory of nonlinear systems but may be applied to both linear and nonlinear systems. Several techniques attempting to assess complexity have been developed such as detrended fluctuation analysis [2], Lempel-Ziv complexity [3], Lyapunov exponents [4], the correlation dimension (*D*_2_) [5], and approximate and sample entropies [6].

The reduction of HRV complexity has been associated with age, disease, and unbalanced cardiovascular regulation [7]. Complexity measures have been proven to characterize HRV signals more successfully than linear approaches in certain applications [8]. In [9–11] point correlation dimension of HRV signals predicted hypotension events in pregnant women during spinal anesthesia for cesarean section, something which time and frequency domain indices were unable to do.

While these measurements are of considerable interest, their application to HRV has some pitfalls that could mislead their interpretation. One of such limitations arises from their application to limited time series. Correlation dimension estimation is highly dependent on the length of the time series [12]. Several studies have reported the effect of data length on *D*_2_ estimation, as well as proposals to alleviate this effect [13, 14]. Stationarity is another requirement that a time series has to fulfil to obtain reliable results. However, satisfying the constraint of finite series and stationarity at the same time is usually difficult [15]. Yet another limitation of these measurements is the long computational time required. Data length exponentially increases the computational time cost in a classical sequential approach. In the case of *D*_2_, several attempts to try to reduce this factor have been reported by Widman et al. [16] and Zurek et al. [17], the latter proposing parallel computing using MPI (Message Passing Interface).

The main goal of this study is to propose a methodological framework for robust and fast estimation of *D*_2_ and its application in HRV signals.  Section 2 starts with a definition of the correlation dimension and its classical estimation. An algorithm for its fast computation is proposed. Robustness is addressed by fitting the log-log curve to a sigmoid function after which three alternative approaches for *D*_2_ estimation are presented.  Section 3 introduces synthetic and real (HRV signals) data where the proposed estimates are evaluated and interpreted.  Section 4 presents the results while Section 5 sets out the discussion and conclusions of the study.

## 2. Methods

### 2.1. Correlation Dimension

Let *x*(*n*), *n* = 1,…, *N* be the time series of interest, which in HRV analysis will be the *RR* interval series normalized to unit amplitude, with *N* being the total number of beats. A set of *m-*dimensional vectors, **y**_*m*_(*i*), called reconstructed vectors, are generated [18]:

(1) $$\begin{array}{c} y_{i}^{m}={\left[x(i),x(i+\tau ),x(i+2\tau ),\ldots ,x(i+(m-1)\tau )\right]}^{T}, \end{array}$$

where *τ* represents the delay between consecutive samples in the reconstructed space. Then, the amount of reconstructed vectors is *N*_*m*_ = *N* − *τ*(*m* − 1) for each *m-*dimension. The distance between each pair of reconstructed vectors, **y**_*i*_^*m*^, **y**_*j*_^*m*^, is denoted as

(2) $$\begin{array}{c} d_{i,j}^{m}=d\left(y_{i}^{m},y_{j}^{m}\right) \end{array}$$

and this can be computed as the norm of the difference vector Δ**y**_*i*,*j*_^*m*^ = **y**_*i*_^*m*^ − **y**_*j*_^*m*^. (In Appendix A different norms and their effect on correlation dimension estimates from finite time series are discussed.) The correlation sum which represents the probability of the reconstructed vector pair distance being smaller than a certain threshold *r* is computed as

(3) Cm(r)=1Nm(Nm−1)∑i,j=1NmH(r−di,jm)=1Nm(Nm−1)∑i=1Nmcim(r),

where *H*(·) is the Heaviside function defined as:

(4) H(x)={1,x≥0,0,x<0,

and *c*_*i*_^*m*^(*r*) = ∑_*j*=1_^*N*_*m*_^ *H*(*r* − *d*_*i*,*j*_^*m*^).

For deterministic systems, *C*_*m*_(*r*) decreases monotonically to 0 as *r* approaches 0, and it is expected that *C*_*m*_(*r*) is well approximated by *C*_*m*_(*r*) ≈ *r* ^*D*_2_^*m*^^. Thus, *D*_2_^*m*^ can be defined as

(5) $$\begin{array}{c} D_{2}^{m}=\underset{r\to 0}{\lim}\frac{\log C_{m}\left(r\right)}{\log\left(r\right)}. \end{array}$$

For increasing *m*, *D*_2_^*m*^ values tend to saturate to a value *D*_2_ which constitutes the final correlation dimension estimate.

### 2.2. Fast Computation of Correlation Sums

One important limitation of *D*_2_ estimation is the long computational time required mainly due to the sequential estimation of correlation sums. This section describes an algorithm for the fast computation of correlation sums based on matrix operations (MO). A matrix **S** which contains the differences between all pairs of samples of *x*(*n*) is computed as

(6) $$\begin{array}{c} S=X-X^{T}, \end{array}$$

where **X** is the *N* × *N* matrix:

(7) $$\begin{array}{c} X=\left(\begin{array}{cccc} x\left(1\right) & x\left(2\right) & \cdots & x\left(N\right)\\ x\left(1\right) & x\left(2\right) & \cdots & x\left(N\right)\\ x\left(1\right) & x\left(2\right) & \cdots & x\left(N\right)\\ \vdots & \vdots & \ddots & \vdots \\ x\left(1\right) & x\left(2\right) & \cdots & x\left(N\right) \end{array}\right), \end{array}$$

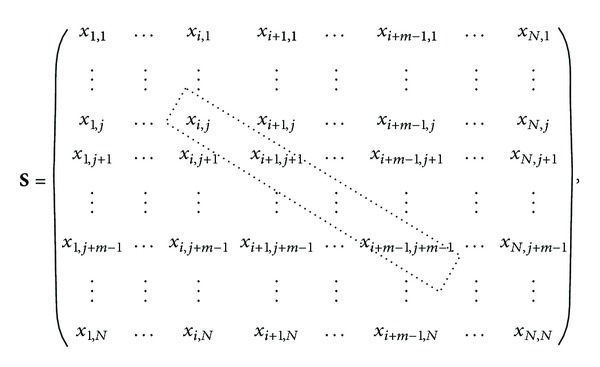
(8)
where *x*_*i*,*j*_ symbolizes *x*(*i*) − *x*(*j*). For instance, the dashed box contains the elements of the difference vector Δ**y**_*i*,*j*_^*m*^ for *τ* = 1. For each embedded dimension *m* and the reconstructed vector *i*, the difference vectors Δ**y**_*i*,*j*_^*m*^ generates a **S**_*i*_^*m*^ matrix:

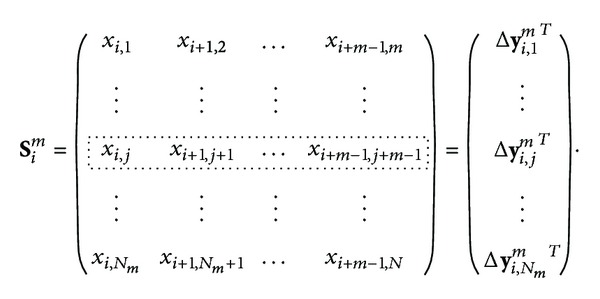
(9)


The selected norm is applied to the matrix **S**_*i*_^*m*^, generating the norm vector **d**_*i*_^*m*^, whose elements are distances **d**_*i*,*j*_^*m*^. To compute the limit in (5), distances should be compared with a set of thresholds, which implies the repetition of the whole process as many times as the number of thresholds. This repetition is avoided since distances in **d**_*i*_^*m*^ are compared with a whole set of thresholds **r** = [*r*_1_, *r*_2_,…, *r*_*N*_*r*__]:

(10) $$\begin{array}{c} \Gamma_{i}^{m}=\left(\begin{array}{cccc} H\left(r_{1}-d_{i,1}^{m}\right) & H\left(r_{2}-d_{i,1}^{m}\right) & \cdots & H\left(r_{N_{r}}-d_{i,1}^{m}\right)\\ H\left(r_{1}-d_{i,2}^{m}\right) & H\left(r_{2}-d_{i,2}^{m}\right) & \cdots & H\left(r_{N_{r}}-d_{i,2}^{m}\right)\\ \vdots & \vdots & \vdots & \vdots \\ H\left(r_{1}-d_{i,N_{m}}^{m}\right) & H\left(r_{2}-d_{i,N_{m}}^{m}\right) & \cdots & H\left(r_{N_{r}}-d_{i,N_{m}}^{m}\right) \end{array}\right), \end{array}$$

where Γ_*i*_^*m*^ is a *N*_*m*_ × *N*_*r*_ matrix, which contains ones and zeros. The accumulative addition of each column represents the partial correlation sum of the *i*th reconstructed vector for a set of thresholds *r*:

(11) $$\begin{array}{c} c_{i}^{m}={\Gamma_{i}^{m}}^{T}1=\left(\begin{array}{c} c_{i}^{m}\left(r_{1}\right)\\ c_{i}^{m}\left(r_{2}\right)\\ \vdots \\ c_{i}^{m}\left(r_{N_{r}}\right) \end{array}\right), \end{array}$$

where 1 is a *N*_*m*_ × 1 vector whose elements are equal to one.

Finally, the procedure has to be repeated varying the *i* index *N*_*m*_ times to compute *C*_*m*_(*r*). This technique saves computational time due to the usage of a set of thresholds in one step.

### 2.3. New Approaches for *D*_2_ Assessment

#### 2.3.1. Sigmoid Curves as Surrogates of Log-Log Curves

*D*_2_ has to be estimated from (5) whose numerator and denominator both tend to −*∞* as *r* tends to 0. Therefore, applying L'Hôpital's rule the equation can be rewritten as [19]

(12) $$\begin{array}{c} D_{2}^{m}=\underset{r\to 0}{\lim}\frac{\partial \log C_{m}\left(r\right)}{\partial \log\left(r\right)}. \end{array}$$

**Figure 1:**
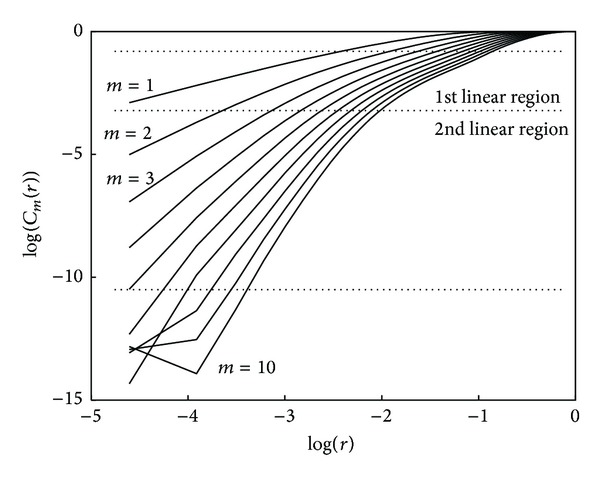
Log-log curves for a dynamic system. Data correspond to an RR interval series extracted from 30 mins of ECG recording.

Since the size of the time series is finite, choosing small values of *r* to evaluate this limit is problematic. For values of *r* close to 0, very few distances contribute to the correlation sum, making the estimation unreliable. The evaluation of this expression is usually done in a linear region in the log⁡(*C*_*m*_(*r*)) versus log⁡(*r*) representation, called the log-log curve. The slope of this linear region is considered an estimate of *D*_2_^*m*^. There are different approaches for estimating this slope. Maximum slope searching can be done by directly computing the increments in the log-log curve. Another approach is to estimate numerically the maximum of the first derivative of the log-log curve. Nevertheless these approaches encounter some limitations due to the usual nonequidistant sampling of *r* values in the logarithmic scale. Yet another limitation arises in the presence of dynamic systems whose log-log curves display several linear regions, as can be seen in Figure 1 where the data corresponds to an RR interval series extracted from a 30 minute ECG recording. In order to estimate the slope of the linear region of the log-log curve, an attempt to artificially extend the linear region is made by excluding the self-comparisons (*d*_*i*,*i*_) from the correlation sums.

**Figure 2:**
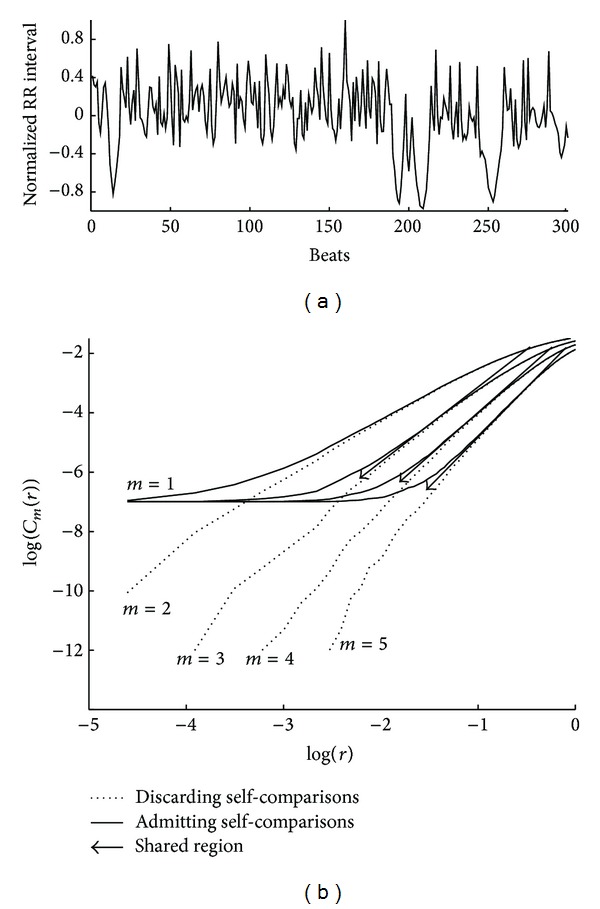
Log-log curves discarding and accepting self-comparisons. Arrows show the slope of the scaling range. Data correspond to an RR interval series of 300 beats.

However, the basis of the approach proposed in this work to improve *D*_2_ estimation lies in considering self-comparisons.  Figure 2 illustrates how log-log curves behave in both situations, considering or not considering self-comparisons. As it is shown, both share part of the linear region. Our proposal is to use sigmoidal curve fitting (SCF) over the log-log curves to obtain an analytic function whose maximum slope in the linear region is well defined. These log-log curves are reminiscent of the biasymptotic fractals studied by Rigaut [20] and Dollinger et al. in [21] in which exponential fittings were proposed. The sigmoidal fitting is applied to the interpolated log-log curves computed with evenlyspaced *r* values.

A modified Boltzmann sigmoid curve was used by Navarro-Verdugo et al. [22] as a model for the phase transition of smart gels:

(13) $$\begin{array}{c} f\left(x\right)=A_{2}-\frac{\left(A_{2}-A_{1}\right)}{B+e^{\left(-x+x_{o}\right)/\alpha }}, \end{array}$$

where *A*_1_, *A*_2_, *x*_*o*_, and *B* are the parameter designed. The first derivative of *f*(*x*) is

(14) $$\begin{array}{c} \frac{df\left(x\right)}{dx}=-\frac{\left(A_{2}-A_{1}\right)e^{\left(-x+x_{o}\right)/\alpha }}{\left[\alpha {\left(B+e^{\left(-x+x_{o}\right)/\alpha }\right)}^{2}\right]}. \end{array}$$

In our study the sigmoid curve *f*(*x*) is fitted to log-log curve. The first derivative, (14), is determined analytically and its maximum constitutes the slope of the linear range, that is, ${\hat{D}}_{2}^{m}$. Note that hat notation refers the use of SCF.

In order to achieve a good fitting, the thresholds, **r**, have to guarantee that both asymptotes are reached. In this work **r** ∈ [0.01   3] with a step of 0.01. The upper asymptote is reached when all comparisons are above the threshold, $\begin{array}{c} C_{m}(r)\approx 1 \end{array}$, and the lower when only the self-comparison is below, *C*_*m*_(*r*) = 1/*N*_*m*_(*N*_*m*_ − 1).

The SCF approach is robust in the presence of dynamic systems which exhibit log-log curves with more than one linear region since when the fitting is not good enough, no estimate is given. In this work, the requirement for a good fitting is to achieve a regression factor greater than 0.8.

As the embedding dimension *m* increases, the linear regions of the log-log curves tend to be parallel to each other. Thus, ${\hat{D}}_{2}^{m}$ estimates tend to saturate to a certain value, which is considered the correlation dimension ${\hat{D}}_{2}$. The correlation dimension is estimated fitting the ${\hat{D}}_{2}^{m}$ versus *m* curve following a modified version of that used by Carvajal et al. [23]:

(15) $$\begin{array}{c} D_{2}^{m}=D_{2}\left(1-Ae^{-km}\right), \end{array}$$

where *A*, introduced in this study in order to reach the saturation level more quickly than previously proposed, and *k* are exponential growth factors.

#### 2.3.2. New Approaches for *D*_2_ Estimation

As mentioned previously, we chose ${\hat{D}}_{2}^{m}$ as the maximum slope on each fitted sigmoid curve. Nevertheless the linear range is composed of more than one point. Instead of considering only one point per curve, in this study we propose a new approach for *D*_2_ estimation considering a set of points extracted from these linear ranges.

**Figure 3:**
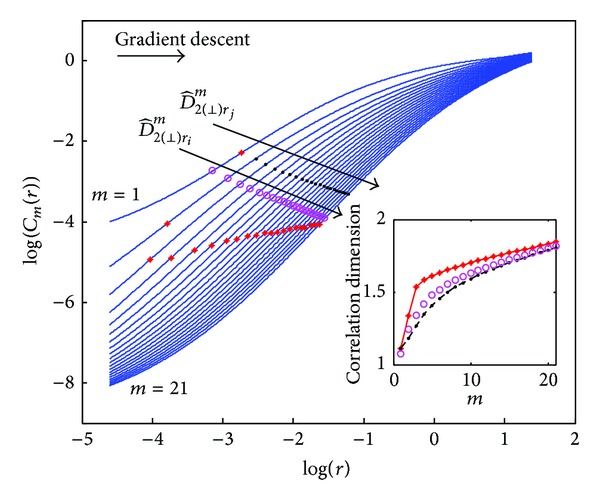
Maximum slope points are marked with crosses over fitted sigmoid curves. Points calculated using gradient descent criteria from two starting points are shown in dots and circles. *r*_*j*_ is the point which corresponds to the maximum slope in the lowest embedded dimension. The inset illustrates the correlation dimension estimation of the three sets of points.

The proposed strategy is based on selecting one point of the linear range in the SCF log-log curve of the lowest embedded dimension *m* and moving forward to the next embedded dimension *m* + 1, selecting the point of the corresponding SCF log-log curve with minimum distance to the former curve (i.e. where the perpendicular to the *m*th log-log curve intersects the (*m* + 1)th log-log curve, as in the gradient descent technique); see Figure 3. The procedure is repeated up to the maximum embedded dimension analyzed. Then, several sets of slopes are computed (one for each point in the linear region around the maximum slope of the SCF log-log curve of the lowest embedded dimension) providing a set of correlation dimension estimates per embedded dimension (${\hat{D}}_{2(⊥),r}^{m}$). The dependence on *r* in the notation indicates that each set of correlation dimension estimates is linked to an *r* value, corresponding to the first value of each set.

Finally, (15) is used to estimate the final correlation dimension (${\hat{D}}_{2(⊥),r}$) for each set of points. These (${\hat{D}}_{2(⊥),r}$) estimates are linked to the log⁡(*r*) value of the lowest embedded dimension. Finally, the maximum of the ${\hat{D}}_{2(⊥),r}$ is selected as the new *D*_2_ estimate, called ${\hat{D}}_{2(⊥)}$.

**Figure 4:**
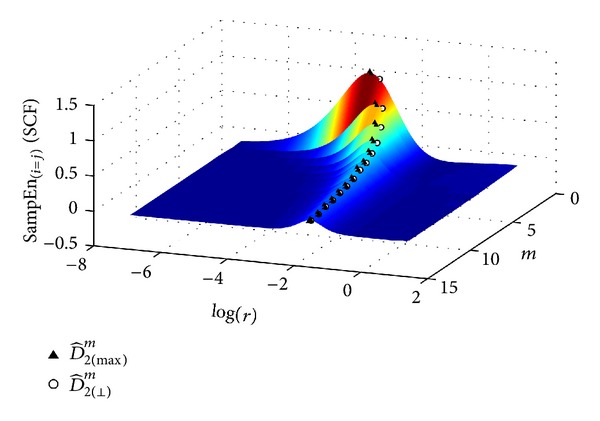
SampEn_*i*=*j*_(*m*, *r*) surface for a 300-beat RR interval series. For each embedded dimension maximum point is marked with solid triangle. Circles correspond to the *r* values which define ${\hat{D}}_{2(⊥)}$.

Another new approach for *D*_2_ estimation based on sample entropy (SampEn) is now presented. SampEn was defined by Zurek et al. [17] as

(16) $$\begin{array}{c} \text{SampEn}\left(m,r,N\right)=\log\left(C_{m}\left(r\right)\right)-\log\left(C_{m+1}\left(r\right)\right), \end{array}$$

where, in this case, *C*_*m*_(*r*) is computed as in (3), but without considering self-comparisons. Let us define SampEn_*i*=*j*_(*m*, *r*) as the sample entropy considering self-pairs, which is easily computed for all embedded dimensions *m* and a huge set of thresholds **r** using the fast algorithm described in Section 2.2. We can generate a SampEn_*i*=*j*_(*m*, *r*) surface from the fitted sigmoid curves, as can be seen in Figure 4, an example of a 300-beat RR interval series extracted from one recording of the database used in [10]. For each embedded dimension, the value of *r* which maximizes SampEn_*i*=*j*_(*m*, *r*) is used to evaluate the slope of the linear region of the SCF log-log curves, ${\hat{D}}_{2(\max)}^{m}$, yielding another *D*_2_ estimate, called in this paper ${\hat{D}}_{2(\max)}$.

## 3. Materials

The selected time series chosen to validate the approaches proposed in this paper to estimate *D*_2_ are the Lorenz attractor, the MIX(*P*) process, and real HRV signals, respectively.

*Lorenz Attractor.* The Lorenz system is described by three coupled first order differential equations whose solution exhibits a chaotic behaviour system for certain parameter values and initial conditions. This is called the Lorenz attractor:

(17) dxdt=σ(y−x),dydt=ρx−y−xz,dzdt=−βz+xy.

For parameter values *σ* = 10, *ρ* = 28, and *β* = 8/3 the theoretical *D*_2_ value is 2.02 [24]. In this study, the system equations are discretized with a time step of 0.01.

*MI* *X*(*P*)* Signals. *MIX(*P*) is a family of stochastic processes that samples a sine for *P* = 0 and becomes more random as *P* increases (*P* = 1 completely random) [5] following the expression

(18) $$\begin{array}{c} \text{MIX}{\left(P\right)}_{j}=\left(1-Z_{j}\right)X_{j}+Z_{j}Y_{j}, \end{array}$$

where $X_{j}=\sqrt{2}\sin(2\pi j/12)$, *Y*_*j*_ ≡ *i*.*i*.*d* uniform random variables on $\left[-\sqrt{3},\sqrt{3}\right]$, and *Z*_*j*_ ≡ *i*.*i*.*d*. random variables, with *Z*_*j*_ = 1 with probability *P*, and *Z*_*j*_ = 0 with probability 1 − *P*. MIX indicates a mixture of deterministic and stochastic components.

*HRV Signals.* The HRV representation used in this study is the time difference between the occurrences of consecutive normal heart beats, the so-called RR interval. Ectopic beats as well as missed and false detections introduce some extra variability in the RR interval series which is not representative of the ANS activity. Thus, they were detected and corrected [25]. The RR interval series analysed in this study belongs to a database recorded at the Miguel Servet University Hospital in Zaragoza (Spain). That database was used to predict hypotension events during spinal anesthesia in elective cesarean delivery by HRV analysis [10]. It consists of ECG signals from 11 women with programmed cesarean section recorded at a 1000 Hz sampling frequency immediately before the cesarean surgery. Five of them suffered a hypotension event during the surgery (Hyp) and 6 did not (NoHyp). The series analysed correspond to 5 minutes in a lateral decubitus position. See [10] for further database details.

## 4. Results

All the results presented in this section are computed using the *ℓ*_*∞*_-norm. The effect of different norms in *D*_2_ estimation is discussed in Appendix A.

The computational time cost of the correlation sums depends on the length of the data, the maximum embedded dimension considered, and the amount of thresholds used. The results shown in Table 1 correspond to computational time required for different data lengths of Lorenz attractor series, a maximum embedded dimension of 16, and a set of 300 threshold values *r* evenlyspaced from 0.01 to 3. The computational time required for a sequential approach is denoted by *T*_Seq_ whereas the time required for the proposed technique, based on matrix operations, is denoted by *T*_MO_. This allows defining the speed up achieved by the novel approach as the ratio between both measurements, *S*_*p*_ = *T*_Sep_/*T*_MO_. As it is shown in Table 1, *S*_*p*_ increases with data length. For a 300-sample data (usual length for a 5 minute RR interval series) correlation sums are estimated in approximately 1 s.

**Figure 5:**
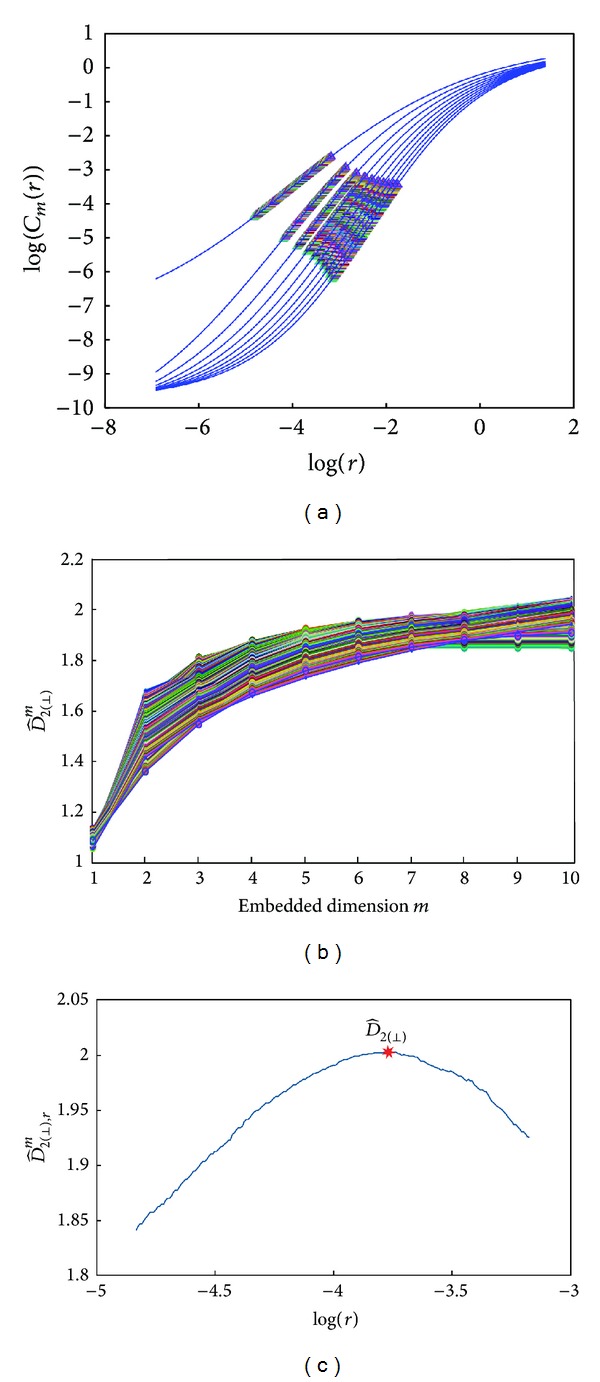
(a) Set of points where slope is estimated from the fitted sigmoid curves in the approach proposed in Section 2.3. (b) Set of ${\hat{D}}_{2(⊥),r}^{m}$ estimates for different starting points versus embedded dimensions are fitted by the exponential equation (15). (c) Correlation dimension estimate for each set corresponding to different starting points. Data extracted from Lorenz attractor of 5000-sample length.

The Lorenz attractor series was used to validate the new proposed methodologies.  Figure 5(a) displays the SCF log-log curves for embedded dimensions *m* from 1 to 10. The sets of points where the slope is evaluated according to (15) are displayed for different starting points. For each starting point, the corresponding set of points is selected following a gradient descent technique.  Figure 5(b) shows the slope estimate (${\hat{D}}_{2(⊥),r}^{m}$) versus *m* for each starting point.  Figure 5(c) displays the correlation dimension estimate (${\hat{D}}_{2(⊥),r}$) versus log⁡(*r*) for each starting point. The maximum (${\hat{D}}_{2(⊥),\;r}$) constitutes the novel *D*_2_ estimate (${\hat{D}}_{2(⊥)}$).

Table 2 displays correlation dimension estimates using the different approaches presented in this study. Note that although the three approaches give results close to the theoretical value of Lorenz attractor correlation dimension, the ${\hat{D}}_{2(⊥)}$ approach is the closest one. Relative errors for approaches ${\hat{D}}_{2}$ and ${\hat{D}}_{2(\max)}$ are above 4%, while for ${\hat{D}}_{2(⊥)}$ it is just 1%; *D*_2_ estimated as described in [10] is also included for comparison purposes.

**Figure 6:**
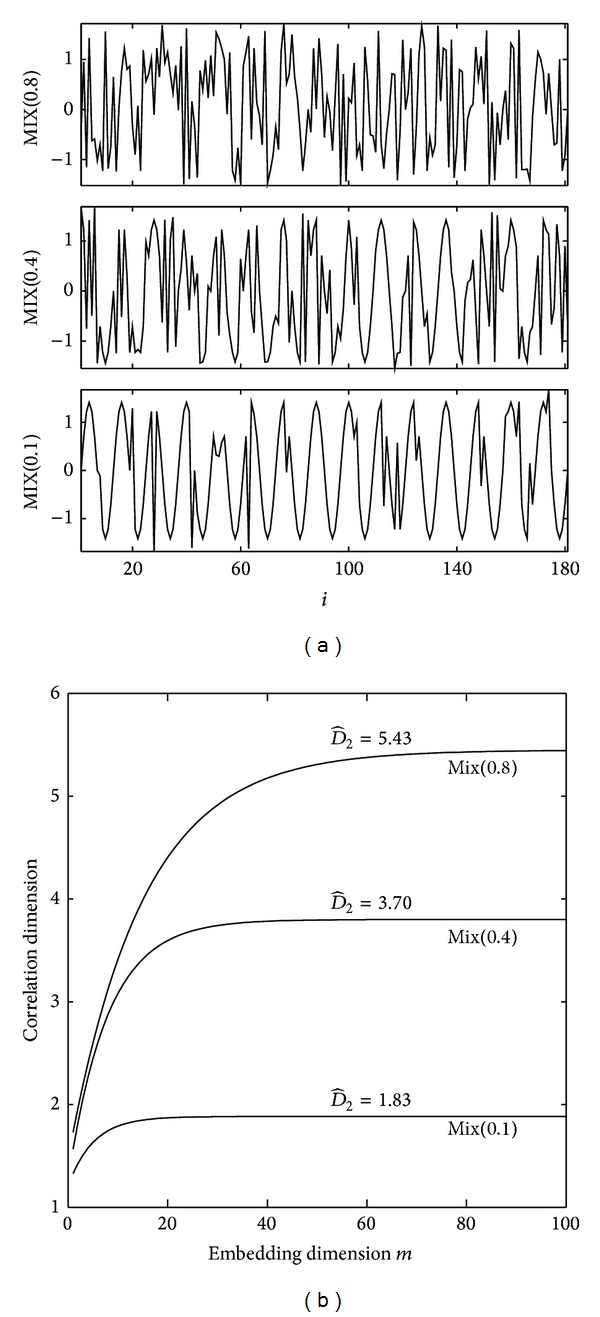
MIX signals with different degrees of randomness and the effects on the estimation of the ${\hat{D}}_{2}$.

${\hat{D}}_{2}$ was applied to a set of MIX series with different *P* values (0.1, 0.4, and 0.8). These estimates can be considered as measures of the randomness of the signals when these signals are finite stochastic processes; see Figure 6.

The same database for HRV analysis as in [10] was used. The results shown in Table 2 are divided into hypotension and nonhypotension groups. The approaches proposed in this paper were applied as well as the classical correlation dimension estimate described in [10] included for comparison purposes. The distribution of the data was found to be not normally distributed by the Kolmogorov-Smirnov test, and therefore the Mann-Whitney *U* test was applied to evaluate their statistical differences in medians. The differences between both groups for all estimates were found to be statistically significant with a *P* value lower than 0.03. In order to evaluate the discriminant power of the proposed measures, ROC analysis was performed. Area of the ROC curve, accuracy, sensitivity, and specificity for all the proposed approaches and the classical *D*_2_ estimate used in [10] were displayed in Table 3. The proposed ${\hat{D}}_{2}$ estimates maintain the accuracy achieved in [10] while the techniques based on the SampEn surface and the gradient descent actually increase it.

## 5. Discussion and Conclusion

In this paper a methodological framework has been proposed to compute the correlation dimension (*D*_2_) of a limited time series such as HRV signals which includes fast computation of the correlation sums, sigmoidal curve fitting of log-log curves, three approaches for estimating the slope of the linear region, and exponential fitting of the ${\hat{D}}_{2}^{m}$ versus *m* curves.

One important limitation for the application of *D*_2_ to HRV analysis is the long computational time required for the correlation sums. In an attempt to solve this problem, an algorithm has been proposed based on matrix operations. In [17] another approach was described based on parallel computing which decreased the time demand. Nevertheless, the computational times achieved in the present work were obtained with a regular computer (Windows 7 based PC, Intel Core i7 3.5 GHz, 16 Gb RAM with Matlab R2011a). As an example, for a signal of 300 sample length (a usual length in typical 5 min HRV analysis, ≈300 beats), the time demand was reduced with respect to the sequential approach from 18 minutes to 1 second, which allows the online computation of *D*_2_ in clinical practice. Computational time required for the proposed approaches is discussed in Appendix B.

Another limitation of the *D*_2_ estimate is its reliability. One of the system characteristics that can lead to an unreliable measurement of *D*_2_ is the nonstationarity of the data. Several techniques attempting to characterize these dynamic systems have been reported, mainly focused on changing the *τ* parameter or even taking into account the time between the vectors [13, 26, 27]. Searching the linear region of the log-log curves becomes a difficult challenge when the system is nonstationary since more than one linear region can appear and classical *D*_2_ estimate is unreliable in those cases. The SCF approach is more robust since it does not give any estimate if the fitting is not good enough.

The novel approaches proposed in this study for the estimation of *D*_2_ use the SCF approach. ${\hat{D}}_{2(⊥)}$ exploits the fact that the linear region of the log-log curves is almost parallel for high embedded dimensions. This allows a set of points surrounding the maximum slope point to be considered, and therefore several correlation dimension estimates are obtained for these starting points. ${\hat{D}}_{2(\max)}$ is based on the differences between two consecutive log-log curves that define the SampEn_*i*=*j*_ surface. This surface showed maximum values for each embedded dimension, *m*, and a specific threshold, *r*, providing another estimation of the correlation dimension. ${\hat{D}}_{2(⊥)}$ was found to be the closest to the theoretical correlation dimension value for the Lorenz attractor series with 5000 size points and for *h* = 0.01, with a relative error of 1%, while ${\hat{D}}_{2}$ and ${\hat{D}}_{2(\max)}$ obtained a relative error of 4% with the same data.

The correlation dimension is known to be a surrogate of the fractal dimension of a chaotic attractor [12]. However, when applied to limited time series, nonzero finite correlation dimension values do not imply the existence of an underlying chaotic attractor. For example, when applied to MIX processes, nonzero finite *D*_2_ values were obtained, higher for more random processes. Thus, although *D*_2_ cannot be interpreted as the fractal dimension of an underlying chaotic attractor, it still gives a measure of the complexity of the process at least regarding its unpredictability.

**Figure 7:**
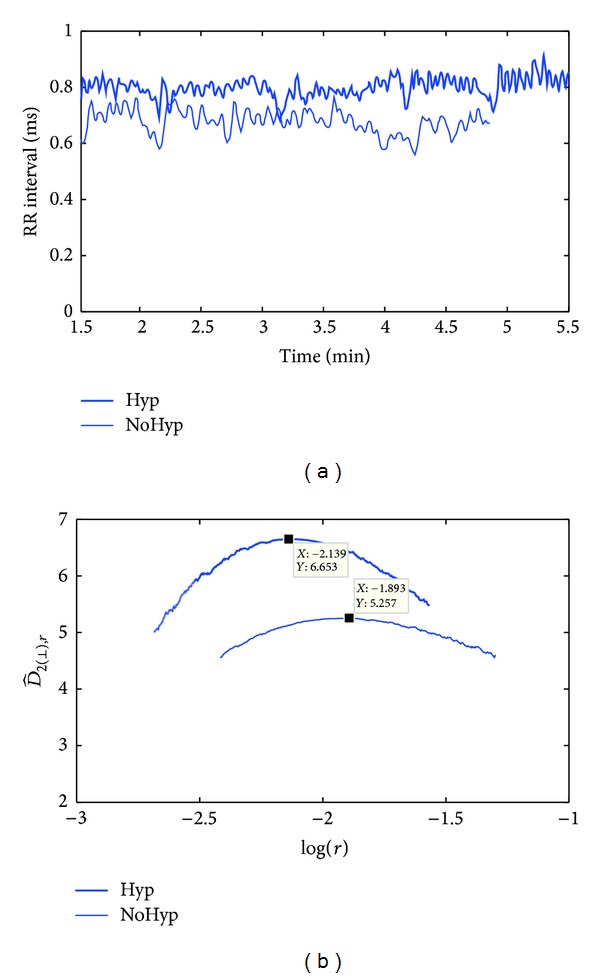
The left panel shows two RR intervals, one corresponding to a patient who developed a hypotension event (Hyp) and the other to one who did not (NoHyp); the right panel shows the ${\hat{D}}_{2(⊥)}$ estimation using the perpendicular points in the log-log curves.

Thus, the *D*_2_ estimate in HRV signals may shed light on the degree of complexity of the ANS or how many degrees of freedom it has. The group of women (Hyp) suffering hypotension events occurring during the surgery of a programmed cesarean section under spinal anesthesia showed higher *D*_2_ values than the group who did not (NoHyp), in the lateral decubitus position. As an example Figure 7 shows one patient of each group and the ${\hat{D}}_{2(⊥)}$ estimate. All the proposed correlation dimension estimates not only maintain the accuracy obtained in [10], they also increase it. Predicting hypotension is a challenge since it occurs in the 60% of the cases producing fetal stress [28]. If the goal is to predict those who are going to suffer hypotension, then the estimates that performed 100% of specificity will be selected, being *D*_2_ [10], ${\hat{D}}_{2}$, and ${\hat{D}}_{2(\max)}$. Otherwise, if the goal is to use prophylaxis in the less number of patients to prevent hypotension, then the estimates that performed 100% of sensitivity will be chosen, and in this case it is ${\hat{D}}_{2(⊥)}$. The effect of prophyilaxis on patients who finally are not going to suffer a hypotension event and the relation with fetal stress needs further studies.

The contribution of this paper to the field is the proposal of a methodological framework for a reliable estimation of the correlation dimension from a limited time series, such as HRV signals, avoiding or at least alleviating the misleading interpretations that can be made from classical correlation dimension estimates. The computational speed-up achieved may allow this framework to be considered for monitoring in clinical practice. Nevertheless, the main limitation for the application of these methodologies to HRV analysis lies in its relation with the underlying physiology, which is still unclear and needs further studies. In spite of the fact that the framework proposed in this paper is focused on the characterization of HRV signals, its applicability could be extended to a wide range of fields. However, an evaluation would be needed to ascertain whether the proposed approaches are appropriate in each particular case.

## Figures and Tables

**Figure 8:**
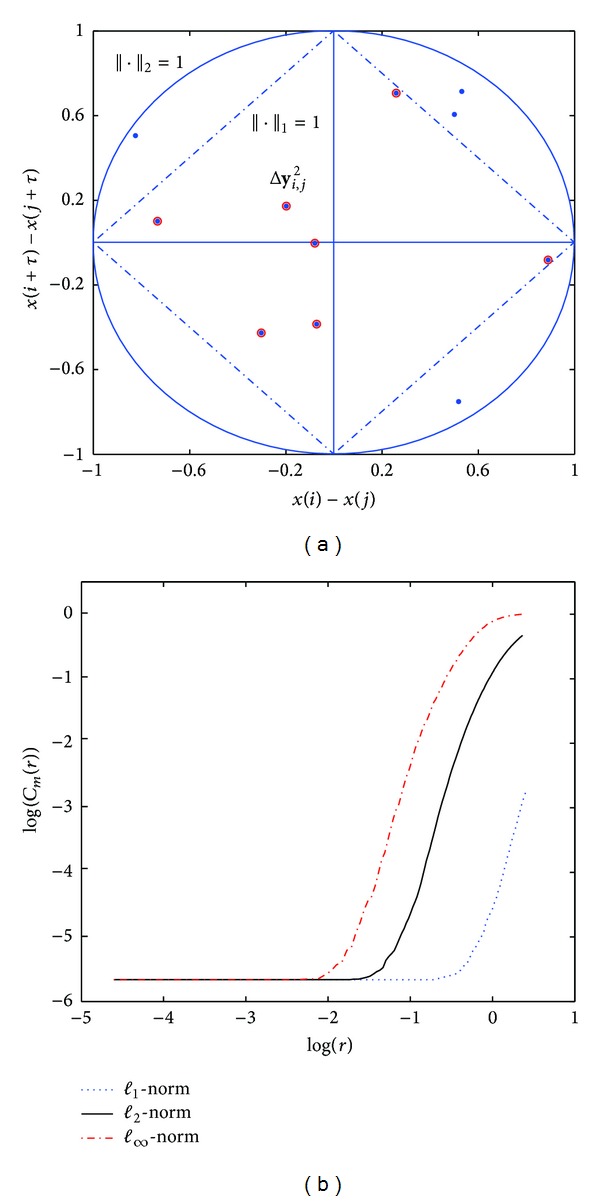
In the left panel, vector differences of any two reconstructed vectors are shown (i.e., for *m* = 2, Δ**y**_*i*,*j*_^2^), where solid circles and dashed lines represent the points whose *ℓ*_2_-norm and *ℓ*_1_-norm are equal to 1, respectively. The dots are the differences below *ℓ*_2_-norm unity and the dots with circles are below *ℓ*_1_-norm unity. In the right panel log-log curves of one HRV signal (300 samples) used in the study are shown for a *m* = 10 and *ℓ*_1_-, *ℓ*_2_- and *ℓ*_*∞*_-norms.

**Table 1:** Computational time of correlation sums estimated for Lorenz attractor series of different sample lengths. *S*_*p*_ is the speed-up achieved and defined as *S*_*p*_ = *T*_Seq_/*T*_MO_, where *T*_Seq_ is the time demand for a sequential algorithm and *T*_MO_ the time demand for the proposed technique based on matrix operations.

*N* (samples)	*T*_Seq_ (s)	*T*_MO_ (s)	*S*_*p*_
300	1086	0.9	*≈*1200
5000	8.69*e*5	50	*≈*16000
10000	3.63*e*6	300	*≈*12000

**Table 2:** *D*_2_ estimated by different approaches for Lorenz attractor series (5000 samples) and HRV signals (300 samples). Data expressed as median ∣ interquartile range.

	Lorenz	HRV		
		Hyp	NoHyp	*P* value
*D*_2_ [10]	1.93	5.92∣0.57	4.39∣1.26	0.028
${\hat{D}}_{2}$	1.93	5.94∣0.60	4.81∣0.79	0.028
${\hat{D}}_{2(⊥)}$	2.01	6.41∣0.65	5.11∣0.77	0.028
${\hat{D}}_{2(\max)}$	1.93	5.88∣0.49	4.83∣0.70	0.010

**Table 3:** ROC area for the analysis of all studied correlation dimension estimates for the database used. Accuracy, sensibility, and specificity estimated with the correspondent cut points are expressed in percentage.

	ROC area	Acc.	Sen.	Spe.
*D*_2_ [10]	0.900	81.8	71.4	100
${\hat{D}}_{2}$	0.900	81.8	71.4	100
${\hat{D}}_{2(⊥)}$	0.900	90.9	100	85.7
${\hat{D}}_{2(\max)}$	0.967	90.9	83.3	100

**Table 4:** Correlation dimension estimates for the different proposed approaches, using different norms for Lorenz attractor series (5000 samples) using different norms.

	Lorenz attractor		
	*ℓ*_1_	*ℓ*_2_	*ℓ*_∞_
*D*_2_ [10]	1.95	1.94	1.93
${\hat{D}}_{2}$	1.69	1.70	1.93
${\hat{D}}_{2(⊥)}$	1.84	1.74	2.01
${\hat{D}}_{2(\max)}$	1.99	1.71	1.93

**Table 5:** Computational time cost for correlation dimension estimates by all proposed approaches considering Lorenz attractor series and HRV signals in which *ℓ*_∞_-norm was applied. Data expressed as mean ± standard deviation.

	Lorenz	HRV
	(5000 samples)	(300 samples)
*T*_*D*_2__ (s) [10]	(8.86 ± 0.35)*e*5	3314 ± 180
$T_{{\hat{D}}_{2}}$ (s)	194 ± 29	4.66 ± 0.58
$T_{{\hat{D}}_{2(⊥)}}$ (s)	260 ± 38	214 ± 30
$T_{{\hat{D}}_{2(\max)}}$ (s)	194 ± 29	4.30 ± 0.56

## Appendices

### A. Use of Norms and Thresholds in Correlation Dimension Estimates

The correlation dimension is considered norm invariant [14]. However, the effect of selecting the norm in correlation dimension estimates deserves further attention when applied to a finite data set. The norm of the difference vector Δ**y**_*i*,*j*_^*m*^ defines the distance *d*_*i*,*j*_^*m*^ in (2). Norms can be defined from *ℓ*_1_ (||·||_1_) to *ℓ*_*∞*_ (||·||_*∞*_). Left panel in Figure 8 shows norm unity for *ℓ*_1_ and for *ℓ*_2_. Moreover, it is illustrated how a distance *d*_*i*,*j*_^2^ can be lower than the norm unity or not depending on which norm is used. The norm unity is chosen as an example of any threshold *r* used in the correlation dimension algorithm. Therefore, by fixing the set of thresholds, the appearance of the linear region of the log-log curve can be compromised.

In Figure 8 the right panel shows how the application of different norms shifts the log-log curves losing the entire linear region in some cases due to the fixed range of thresholds. The range of these thresholds should be long enough to ensure that the linear regions are contained therein; thus, the election of the norm compromises the set of thresholds used.

In the SCF approach it is particularly important that the two asymptotic regions should be represented in the log-log curve. Therefore, the correct selection of the norm and the range of the set of thresholds are critical to assure the goodness of the SCF approach.  Table 4 shows the correlation dimension estimates for 5000 data length of Lorenz attractor series. The effect of different norms is reflected in the estimates since the set of thresholds was fixed. As it is shown, the application of *ℓ*_*∞*_-norm, combined with the fixed set of thresholds used, achieves closest values with respect to the theoretical correlation dimension value for Lorenz attractor, 2.01.

### B. Computational Time Demand of Novel Approaches to Correlation Dimension Estimates

In Section 2.2 a new technique based on matrix operations (MO) was introduced in order to compute correlation sums which represent the core of the correlation dimension algorithm. Nevertheless, in the paper no computational time cost was considered for the new proposed approaches for correlation dimension estimates.  Table 5 shows the time required for the correlation dimension estimates including that used in [10].

10 realizations of Lorenz attractor series were generated whose initial conditions were randomly chosen. It is noticeable that the time cost of ${\hat{D}}_{2(⊥)}$ is higher compared to the others in both cases, the Lorenz series and the HRV signals (11 subjects), since it uses several sets of slope estimated to compute correlation dimension. Furthermore, the ratio between them is higher for the HRV signals than for the Lorenz series. Each of the different sets of thresholds is associated with an *r* value in an interval centred on the maximum slope for *m* = 1. This interval is defined as a decrease in 50% of the amplitude of the maximum in the SCF first derivative. The more abrupt the transition zone in the sigmoid, the lower the amount of starting points. Thus, each realization is done with a different number of points, varying the computational time.
